# Improving Restoration Efficiency by Modeling Optimal Sowing Periods: A Case Study of Two Native Plants and Restoration of a Site

**DOI:** 10.3390/plants10081506

**Published:** 2021-07-22

**Authors:** Young-Ho Jung, JunHyeok Kim, Chung-Youl Park, Hee-Seung Park, YoSup Park

**Affiliations:** 1 Division of Wild Plant Seeds Research, Baekdudaegan National Arboretum, Bonghwa 36209, Korea; jyh5250@kiam.or.kr (Y.-H.J.); kjh9859@kiam.or.kr (J.K.); doonas@kiam.or.kr (C.-Y.P.); 2 Department of Integrative Plant Science, School of Bioresource and Bioscience, Chung-Ang University, Anseong 17546, Korea; 3 Citrus Research Institute, National Institute of Horticultural & Herbal Science, RDA, Jeju 63607, Korea

**Keywords:** *Agastache rugosa*, *Astilbe rubra*, daily mean temperature, daily temperature range, ecological restoration, Mt. Gariwang, seed germination, optimal sowing period

## Abstract

Efficient ecological restoration techniques are urgently required to minimize seed consumption and labor requirements. Here, we determined the optimal sowing period for two native species, *Agastache rugosa* (Korean mint) and *Astilbe rubra* (False goat’s beard), toward their use for ecological restoration of Mt. Gariwang, a site damaged by the 2018 PyeongChang Winter Olympics’ activities. We investigated the effects of daily mean temperature (DMT) and daily temperature range (DTR) on seed germination percentage, which decreased for both species with decreasing DTR and was optimal at a DMT of 25 °C and 17.5 °C for *A. rugosa* and *A. rubra*, respectively. We developed a single multiple regression equation that evaluated the effects of DMT and DTR simultaneously and determined the temperature scores when the germination percentage reached 85%. We applied the developed multiple regression equation by analyzing the temperature data of the restoration site. In addition, precipitation data analysis was added to set the optimal sowing period. As a result, the optimal sowing period for the two species was determined from May 21 to the end of May. This makes it possible to minimize seed consumption and labor requirements when sowing seeds. The model developed herein will be useful not only to guide the ecological restoration of Mt. Gariwang, but also for other regions using site-specific temperature data.

## 1. Introduction

Mt. Gariwang is a representative damaged site that was affected by the installation of the Jeongseon Alpine Center as part of the 2018 PyeongChang Winter Olympics. Prior to the installation of the center, the site supported the largest wild populations of *Abies nephrolepis* (Trautv.) Maxim. and *Taxus cuspidata* Siebold & Zucc. on Mt. Gariwang and contained trees from older age classes; thus, the Mt. Gariwang area was evaluated as having great preservation value [1,2]. After the installation of the alpine center, the site was left in a damaged state due to either ineffective maintenance of the alpine facility or insufficient ecological restoration efforts. As the ski resort was built on a steep slope, the continuous loss of topsoil is a major concern [3].

When restoring damaged sites, native plants are considered high-priority restoration materials because of their great environmental adaptability to the subject site [4]. In particular, many studies have shown that the loss of exposed soil in damaged lands can be minimized by improving vegetation coverage through sowing seeds of herbaceous native plants [5,6]. *Agastache rugosa* (Fisch. & Mey.), Kuntze (Korean mint), and *Astilbe rubra* (Hook. F. & Thomson) (False goat’s beard) are the representative herbaceous native plants of Mt. Gariwang and have been recommended as suitable species for ecological greening because of their extensive ground coverage and subsequent protection against the damaging effects of wind and rain [7,8].

Ecological restoration through sowing of seeds is dependent on effective seed germination. Seed germination is affected by various environmental factors, such as temperature, moisture, and soil composition [9,10]. In particular, seed germination is largely controlled by temperature, among other environmental factors [11,12]. Many studies have investigated the impact of temperature on germination [13,14,15], the results of which can be used to establish the optimal seed sowing period. Sowing period has a significant impact on seed germination and is, therefore, a major determinant of the success of ecological restoration efforts [16,17]. In addition, there are several examples where such data have been applied to plan ecological restoration and to predict plant adaptation to climate change through the scoring of influential temperatures [18].

In this study, we aimed to determine the optimal sowing period of *A. rugosa* and *A. rubra* toward their effective use in the restoration of a site damaged by construction activities related to the 2018 PyeongChang Winter Olympics. For this purpose, we (1) investigated the germination of *A. rugosa* and *A. rubra* seeds under different temperatures and temperature ranges, (2) developed a model that simultaneously assessed the effects of temperature (mean and range) on germination success rates, and (3) determined the optimal sowing period for each of the two species (i.e., for maximized restoration efficiency) by inputting the actual temperature data of Mt. Gariwang in the experimentally developed model. In addition, accuracy was improved by applying precipitation data collected during the rainy season in order to accurately reflect the climate characteristics of Korea.

## 2. Results

### 2.1. Customized Germination Model for A. rugosa and A. rubra Seeds

**Figure 1:**
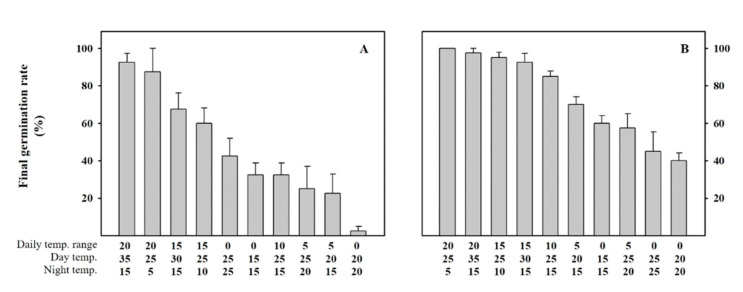
Final germination percentage of *Agastache rugosa* (**A**) and *Astilbe rubra* (**B**) under 10 different temperature conditions. Final germination percentage represents the value on the date when the germination percentage remained unchanged after more than 5 days. The bars represent the mean ± SE (*n* = 4).

As a first step in modeling the germination of *A. rugosa* and *A. rubra* seeds, we examined the effects of the experimental temperature conditions on the germination percentage of *A. rugosa* and *A. rubra* seeds (Figure 1). The final germination percentage of the seeds of both species decreased with a decreasing temperature range (i.e., the temperature difference between the daytime and nighttime temperatures). In particular, for *A. rugosa* seeds, when the temperature difference was lowered to <10 °C, the final germination percentage was <50%. This result indicated that not only does the supply of a specific temperature affect the germination percentage, but also, the daily temperature range (DTR)does. Thus, both daily mean temperature (DMT) and DTR were incorporated in the subsequent modeling process to account for the influence of both mean temperature and temperature difference between daytime and nighttime.

As a second step, the standardized regression coefficient, which is the relative influence of the cumulative temperature on the germination percentage, was obtained through multiple regression analysis (Table 1). Due to the good regression coefficients obtained for all temperature ranges of the cumulative DMT and DTR sets in this study, therefore, analyses were conducted for all temperature conditions (Table 1). The standardized regression coefficient relative to the cumulative DMT did not exhibit a proportional relationship with cumulative DMT for both species. The cumulative DMT corresponding to the highest standardized regression coefficient differed between species: 25 °C for *A. rugosa* and 17.5 °C for *A. rubra*. As a result, the impact weight was converted by setting the standardized regression coefficients of *A. rugosa* at 25 °C and those of *A. rubra* at 17.5 °C to 1.

**Figure 2:**
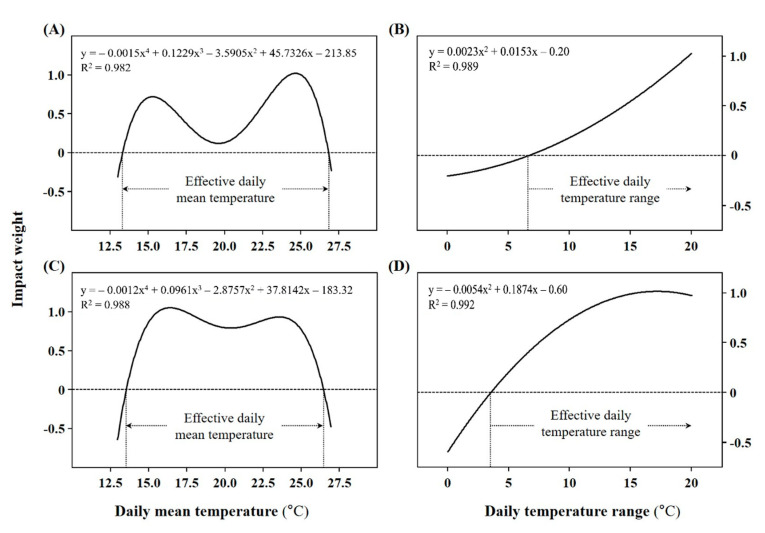
Regression curve of the impact weight on germination under two different temperature conditions of daily mean temperature and daily temperature range. Graphs in panels (**A**,**B**) show the results for *Agastache rugosa* and those in panels (**C**,**D**) show the results for *Astilbe rubra*. The curve was derived using the standardized regression coefficient ratios (*n* = 160).

The cumulative DTR that corresponded to the standardized regression coefficient was the highest at 20 °C for *A. rugosa*. The standardized regression coefficient tended to decrease at temperatures lower than 20 °C. In turn, the highest standardized regression coefficient for *A. rubra* was observed at 15 °C, and the standardized regression coefficient tended to decrease as the temperature deviated from 15 °C. This implies that, among the cumulative DTRs that affect the germination percentage of *A. rugosa* and *A. rubra* seeds, temperatures of 20 and 15 °C, respectively, had the greatest impact on the germination percentage, and that this impact decreases as cumulative DTR deviates from 20 and 15 °C, respectively. Similarly, the impact weight was converted by setting the standardized regression coefficients of *A. rugosa* at 20 °C and of *A. rubra* at 15 °C to 1. A non-linear regression analysis was performed to estimate the influence of different temperatures, including those not included in the experiment. Four equations were derived according to cumulative DMT and DTR and the two species (Figure 2).

The impact weight on germination was evaluated for temperatures at 0.1 °C intervals using the derived equation. For *A. rugosa*, the peaks of the equation were found at DMTs of 15.3, 19.6, and 24.6 °C. The impact of temperature on germination percentage was highest at DMTs of 15.3 and 24.6 °C, and decreased with deviation from these two temperatures. Moreover, DMTs below 13.5 °C and above 26.7 °C and a DTR above 6.7 °C were found to have a negative weight.

As for *A. rubra*, the peaks of the DMT regression equation were found at 16.4, 20.5, and 23.6 °C. The impact of temperature on the germination percentage was highest at DMTs of 16.4 and 23.6 °C and decreased with deviation from these two temperatures. Moreover, DMTs below 14.3 °C and above 25.6 °C and a DTR above 3.6 °C were found to have a negative weight. The effects of each DMT and DTR on germination can be evaluated using the determined weights. The temperature ranges determined as having a negative weight were analyzed to offset the cumulative effect of different temperatures on germination. The above results were consistent with those of the analysis of the actual target site temperatures.

As a final step in modeling the germination of *A. rugosa* and *A. rubra*, we performed a multiple regression analysis on cumulative germination percentage and the cumulative impact weights derived from each equation in Figure 2. Through this, we combined the equation for each species into a single equation, while simultaneously evaluating the DMT and DTR (Table 1 and Table 2). Consequently, equations with coefficients of determination (R^2^) of 0.841 and 0.751 were derived for *A. rugosa* and *A. rubra*, respectively. The resulting value obtained by substituting the DMTs and DTRs into the equation represented the temperature score (TS).

### 2.2. Temperature Requirement for a Final Germination Percentage of 85%

**Figure 3:**
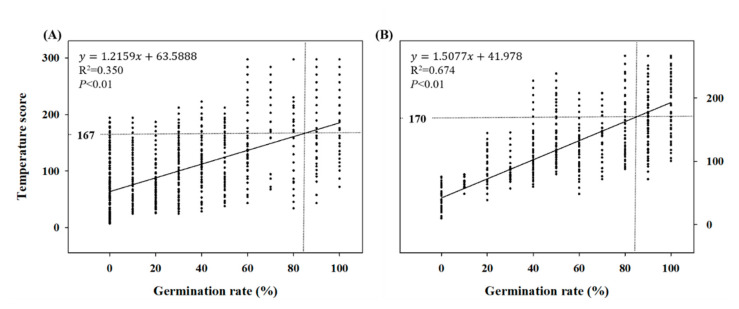
Linear regression analysis between daily temperature score (TS) and germination percentage of *Agastache rugosa* (**A**) and *Astilbe rubra* (**B**). TS is the accumulated value of the daily temperature data according to the equation in Table 2. Temperature requirement is cumulative TS at 85% germination (*n* = 300).

A linear regression analysis of the germination percentage and cumulative TS was performed to measure the temperature requirements of *A. rugosa* and *A. rubra* to achieve a final germination percentage of 85% (Figure 3). The seed germination percentage of *A. rugosa* and *A. rubra* was found to be >85% at cumulative daily temperatures of 167 TS and 170 TS, respectively.

### 2.3. Prediction of the Adaptation of A. rugosa and A. rubra to the Mt. Gariwang Site

We analyzed the temperature data of Mt. Gariwang from 2015 to 2018 to apply the developed multiple regression equation to the target site. In addition, the precipitation data of the target site were also analyzed to improve the reliability of the optimal sowing period. Our results showed that daily maximum temperature, daily minimum temperature, highest DMT, lowest DMT, and average DMT were 35.3, −25.3, 27.3, −20.6, and 7.9 °C, respectively. The highest DTR, the lowest DTR, and the average DMT range were 21.9, 0.8, and 8.6 °C, respectively. The average monthly accumulated precipitation was 15.63, 32.38, 54.38, 120.25, 94.25, 101.75, 348.13, 156.00, 92.25, 85.00, 85.88, and 40.63 mm sequentially from January to December. Thus, the month with the highest rate of precipitation was July and the month with the lowest was January. The reason why the accumulated precipitation of Mt. Gariwang in July is more than three times that of June is probably because July is the rainy season at Mt. Gariwang. In summer in Korea, the rainy season lasts for 30 days, with 40% of the annual precipitation.

**Figure 4:**
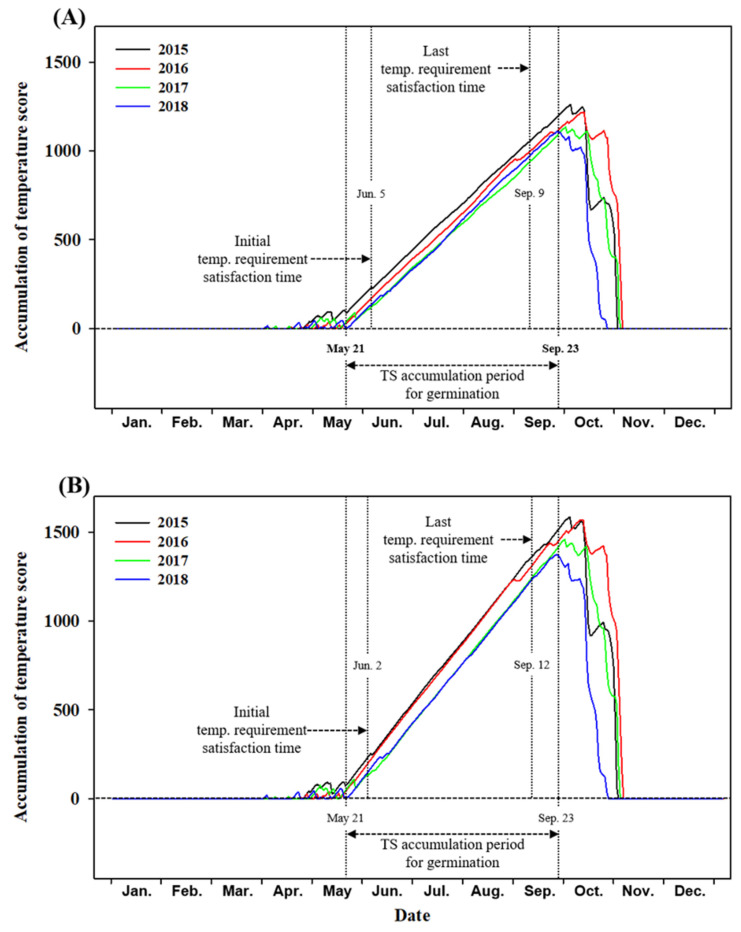
Temperature score (TS) accumulation patterns of *Agastache rugosa* (**A**) and *Astilbe rubra* (**B**) during the 2015–2018 season in Mt. Gariwang, Korea. The TS accumulation period for germination is a common period during 2015–2018 that continuously shows positive effects on germination. Initial temperature requirement is the average time at which TS satisfies the temperature requirement for the first time when sowing before the start of TS accumulation. Last temperature requirement is the last average time to satisfy the temperature requirement. The temperature requirement is 167 TS for *A. rugosa* (**A**) and 170 TS for *A. rubra* (**B**).

The derived germination model for *A. rugosa* and *A. rubra* seeds was applied to the DMT and DTR of Mt. Gariwang from 2015 to 2018 (Figure 4). Commonly, the optimal temperature for germination (effective temperature) of *A. rugosa* and *A. rubra* seeds did not increase to as high as the temperatures required for each species from November to March. In addition, the period in which optimal temperatures were predicted to occur, based on the observations from 2015 to 2018, was from 21 May to 23 September, and this was consistent for *A. rugosa* and *A. rubra.* As a result, the predicted seed germination periods for *A. rugosa* and *A. rubra*, sown during the optimum temperature accumulation period or prior to the period, reached 167 TS and 170 TS, respectively. The temperature requirement for germination of seeds sown in April, and when this temperature requirement would be met, were examined to determine the appropriate sowing period for *A. rugosa* and *A. rubra* seeds. The data showed that, on average, the temperature requirement would expectedly be reached on 5 June and 2 June for *A. rugosa* and *A. rubra*, respectively. Furthermore, the average final dates when the temperature requirements can be met were predicted to be on 9 September and 12 September for *A. rugosa* and *A. rubra*, respectively.

## 3. Discussion

Effective ecological restoration by means of seeds requires elucidating the conditions needed for successful seed germination. Temperature is a major factor affecting seed germination percentage and velocity [11,12]. Furthermore, the temperature factors that affect germination include DMT and DTR. It is difficult to evaluate these factors with just one indicator because the factors may react differently depending on the species, even under the same conditions [19]. The DMT that corresponded to the highest standardized regression coefficient differed between the two species, i.e., 25 °C for *A. rugosa* and 17.5 °C for *A. rubra* (Table 1). Our findings for *A. rugosa* were consistent with a previous study [20], which observed a slightly higher germination percentage at 25 °C than at low temperatures. Similarly, results for *A. rubra* in our study were consistent with a previous study that found that the germination percentage of another *Astilbe* sp., *A. koreana* Nakai, was higher at low temperatures, i.e., 15 and 20 °C, than at relatively high temperatures, between 25 and 30 °C [21].

The germination percentage of *A. rugosa* and *A. rubra* seeds tended to decrease with decreasing DTR, and the temperature was found to have less of an effect on the germination percentage under fixed temperature conditions than under variable temperature conditions (Figure 1, Table 1). Previous studies that assessed seed germination under various temperature conditions showed that the influence of DTR varies depending on the species [22,23,24]. Thus, for species that are significantly affected by DTR, such as *A. rugosa* and *A. rubra*, the influence of temperature should be evaluated in consideration of DMT as well as DTR, especially since the latter is expected to vary considerably under natural conditions. Accordingly, the aim of the present study was to develop a model that considered both DMT and DTR.

In this study, the experimental daytime and nighttime temperatures were used to define the DMT and DTR values, which, in turn, served as indicators of the influence of temperature and temperature variation. These data were used to develop a model that demonstrated germination percentage tendencies that were consistent with those observed under the actual temperature treatments. The resulting germination model was expressed as an equation that was used to calculate the TS values that represent the impact of temperature on germination percentage. The temperature requirement for effective germination, i.e., the biological time required for the percentage of germination to reach 85%, was expressed by a single value called the “cumulative TS”, and was determined to be 167 TS and 170 TS for *A. rugosa* and *A. rubra*, respectively (Figure 3).

A comprehensive survey of the environmental factors of a site must be conducted to effectively restore its ecology [25]. Here, we collected climate data for the target restoration site, Mt. Gariwang, and applied these data to the model constructed. When *A. rugosa* and *A. rubra* seeds were sown in April, the germination period was predicted to be June 5 and 2, respectively, and the final sowing period during which the temperature requirement could be met was predicted to be September 9 and 12, respectively (Figure 4). Although the influence of the temperature condition differs between the two species, the germination timing of the two species in Mt. Gariwang was predicted to be similar. Based on these results, even species with the same germination timing in an area may be differently affected by temperature conditions. Therefore, models that predict species-specific relationships between germination and temperature need to be developed.

Analysis of the temperature data of Mt. Gariwang during 2015–2018 revealed that the temperatures from 21 May to 23 September were predicted to be optimal for the germination of seeds of both species (Figure 4). Although early sowing before 21 May may still result in the germination of some seeds, sowing after 21 May is recommended to minimize the consumption of seeds and to achieve the maximum germination percentage possible, relative to the total number of seeds sown. In addition, the average monthly accumulated precipitation of Mt. Gariwang was three times higher than that of the previous month, and it was determined that July was the rainy season for Mt. Gariwang. In general, during the rainy season when continuous rainfall falls, seedlings cannot withstand the moisture and the initial growth is poor or withered [26]. Therefore, it is necessary to sow the seeds at least one month before the beginning of the rainy season so that the seedlings can settle normally. In the case of sowing after the rainy season, the growing number of days becomes longer as the growing temperature decreases, and normal growth is difficult due to an inappropriate growth temperature and insufficient amount of light due to typhoons [26].

Lee et al. (1999) suggested 23 March to be the optimal annual seeding date for *A. rugosa* in Korea [27]. Hong et al. (2020) predicted that the optimal seeding period would be earlier in the southern part, where *A. rugosa* is mainly grown, but would be after 23 March in the central mountainous area [28]. We determined the optimal sowing period of *A. rugosa* in Mt. Gariwang, a central mountainous area, to be from 21 May to the end of May, one month before the start of the rainy season. No previous studies have investigated the sowing period of *A. rubra*. In this study, this species was predicted to germinate within the same period as that of *A. rugosa* in Mt. Gariwang. Altogether, these findings indicate that optimal seeding periods are site-specific and should be determined by analyzing the climate data of each seeding site.

This study developed a species-specific germination temperature model for two native plants, *A. rugosa* and *A. rubra*. Mt. Gariwang temperature data were then applied to the developed model to predict possible seed germination periods for the two species in Mt. Gariwang and, in turn, their optimal sowing periods. This model can be used to assess temperature data, generate predictions regarding the optimal germination timing of *A. rugosa* and *A. rubra* in Mt. Gariwang and other areas, and improve ecological restoration efficiency by minimizing the number of seeds used while reducing the labor needed. In addition, the reliability of the sowing period was improved by analyzing the accumulated precipitation data of Mt. Gariwang. This is particularly important given the recent popularization of these species as medicinal plants. Integrated models that can analyze various other factors that affect germination and restoration success, in addition to temperature and accumulated precipitation, should be developed in the future.

## 4. Materials and Methods

### 4.1. Seeds and Experimental Conditions

Seeds of *A. rubra* and *A. rugosa* used in this study were collected from the Baekdudaegan National Arboretum (37.007626 N, 128.832047 E) in October 2017. The seeds were dried in a drying room (15 °C, 15% relative humidity) for approximately two weeks and stored (−20 °C, 40% relative humidity) until use. Seed quality was assessed using a soft X-ray test (EMT-F70, Softex Co., Ltd., Tokyo, Japan), and only intact, healthy seeds were used for the experiments. A thermal gradient plate (TGP, ONSOL. Co., Suwon, Korea) was used to create a temperature gradient environment. The photoperiod was fixed at 12 h (light/dark). Based on the known optimal germination temperature of the two species, i.e., 25/15 °C (day/night), we established a range of experimental daytime temperatures from 15 °C (minimum) to 35 °C (maximum) and a range of experimental nighttime temperatures from 5 °C (minimum) to 25 °C (maximum), at 5 °C intervals [29].

### 4.2. Species-Specific Seed Vigor Test

To evaluate the effects of temperature on the germination percentage of *A. rugosa* and *A. rubra* seeds, we investigated their final germination percentage under different temperature conditions. A standardized regression coefficient was then developed for the germination percentage and the DMT and DTR by conducting a multiple linear regression analysis of germination percentage and temperature conditions. To analyze the cumulative effect of DMT and DTR on germination, the cumulative germination percent data were set as the dependent variable and the cumulative DMT and DTR data for each temperature were set as the independent variables. The development of the standardized regression coefficient enabled (1) the removal of dimensional influence, and (2) the quantification of the effects of DMT and DTR on germination. The relative influence represented by the standardized regression coefficient is converted based on the highest standardized regression coefficient. It was set as an impact weight that expresses the cumulative influence according to each temperature condition. Then, a non-linear regression analysis was performed using the impact weight (the converted standardized regression coefficient). We selected the equation (model) with the simplest structure and an R^2^ value > 0.98 within the range of recognized significance.

Subsequently, a multiple regression analysis was performed on the derived impact weight and germination percentage to simultaneously evaluate DMT and DTR, and the previously derived equations for each species were then combined to form a single equation. Multiple regression analysis was performed by setting the cumulative germination percentage as the dependent variable and the cumulative impact weight derived through the previous nonlinear regression analysis as the independent variable. The output values from inputting DMT and DTR data into the equation were referred to as the daily temperature score (TS). Finally, we investigated the temperature requirements for the germination of *A. rugosa* and *A. rubra*, i.e., the cumulative TS value required for *A. rugosa* and *A. rubra* seeds to reach a germination percentage of 85%, following the seed vitality standard proposed by the Royal Botanic Gardens, Kew, England [30]. For statistical analyses, we used data gathered up to 16 days after sowing, which was the day when the germination percentage remained unchanged for more than 5 days. Statistical analyses were conducted using SPSS 23 (SPSS Inc., Chicago, IL, USA).

### 4.3. Application of Species-Specific Seed Vigor Test for Regional Climate Data

To predict the cumulative TS values and germination period of *A. rugosa* and *A. rubra* seeds on Mt. Gariwang, PyeongChang, we used daily temperature and monthly accumulated precipitation data from the target restoration site available from 2015 to 2018 [31]. The temperature data were applied to the previously developed germination model of *A. rugosa* and *A. rubra* seeds, which revealed the changes in germination percentage in response to the changes in annual temperature. Precipitation is one of the important factors to be considered in setting the seed sowing period [26]. Therefore, by using the analyzed cumulative precipitation data, the reliability of the optimal sowing period for *A. rugosa* and *A. rubra* was improved.

## Figures and Tables

**Table 1:** Multiple regression analysis of the cumulative germination percentage and the cumulative daily mean temperature (DMT) and daily temperature range (DTR) in *Agastache rugosa* and *Astilbe rubra*. The results of all regression analyses showed that the *p*-value was less than 0.001 through the F-test. “Impact weight” was converted based on the largest absolute value of the standardized regression coefficients. The minus regression coefficient means a negative linear relationship between the cumulative germination percentage and the cumulative DTR. (* *p* < 0.05; ** *p* < 0.01; *n* = 160).

Temperature (°C)		*Agastache rugosa*		*Astilbe rubra*	
		Standardized Regression Coefficient	Impact Weight (Ratio)	Standardized Regression Coefficient	Impact Weight (Ratio)
Daily mean temperature (DMT)	15.0	0.488 **	0.71	0.685 **	0.86
	17.5	0.257 **	0.37	0.799 **	1.00
	20.0	0.089 *	0.13	0.640 **	0.80
	22.5	0.421 **	0.61	0.712 **	0.89
	25.0	0.689 **	1.00	0.629 **	0.79
Daily temperature range (DTR)	0	−0.157 **	−0.17	−0.525 **	−0.60
	5	−0.140 **	−0.15	0.179 *	0.20
	10	0.199 **	0.21	0.635 **	0.72
	15	0.538 **	0.58	−0.880 **	1.00
	20	0.929 **	1.00	−0.850 **	0.96

**Table 2:** Multiple regression analysis of the cumulative germination percentage and the cumulative impact weight of daily mean temperature (DMT) and daily temperature range (DTR) in *Agastache rugosa* and *Astilbe rubra*. The values obtained using these equations were set to a temperature score (TS) (*n* = 160).

Species	Variable		Standardized Regression Coefficient	Equation	*p*-Value	R^2^
*Agastache rugosa*	Impact weight of	daily mean temperature (x_1_)	0.409	2.948·x_1_ + 4.041·x_2_ + 7.299	<0.001	0.841
		daily temperature range (x_2_)	0.678			
*Astilbe rubra*	Impact weight of	daily mean temperature (x_3_)	0.613	3.584·x_3_ + 1.679·x_4_ + 7.816	<0.001	0.751
		daily temperature range (x_4_)	0.466			

## Data Availability

Not Applicable.
